# Granulocyte Colony-Stimulating Factor Reduces Fibrosis in a Mouse Model of Chronic Pancreatitis

**DOI:** 10.1371/journal.pone.0116229

**Published:** 2014-12-31

**Authors:** Wey-Ran Lin, Tzung-Hai Yen, Siew-Na Lim, Ming-Der Perng, Chun-Yen Lin, Ming-Yo Su, Chau-Ting Yeh, Cheng-Tang Chiu

**Affiliations:** 1 Department of Gastroenterology, Linkou Chang Gung Memorial Hospital, Taoyuan, Taiwan; 2 Liver Research Unit, Linkou Chang Gung Memorial Hospital, Taoyuan, Taiwan; 3 College of Medicine, Chang Gung University, Taoyuan, Taiwan; 4 Department of Nephrology, Linkou Chang Gung Memorial Hospital, Taoyuan, Taiwan; 5 Department of Neurology, Linkou Chang Gung Memorial Hospital, Taoyuan, Taiwan; 6 Institute of Molecular Medicine, National Tsing Hua University, Hsinchu, Taiwan; University of California, Merced, United States of America

## Abstract

**Background:**

Chronic pancreatitis (CP) is a necroinflammatory process resulting in extensive pancreatic fibrosis. Granulocyte colony-stimulating factor (G-CSF), a hematopoietic stem cell mobilizer, has been shown to exert an anti-fibrotic effect partly through the enrichment of bone marrow (BM) cells in fibrotic organ. We aimed to test the effect of G-CSF on fibrosis in a mouse model of CP.

**Methods:**

CP was induced in C57Bl/6J mice by consecutive cerulein injection (50 µg/kg/day, 2 days a week) for 6 weeks. Mice were then treated with G-CSF (200 µg/kg/day, 5 day a week) or normal saline for 1 week, and sacrificed at week 7 or week 9 after first cerulein injection. Pancreatic histology, pancreatic matrix metallopeptidase 9 (MMP-9), MMP-13 and collagen expression were examined. Pancreatic myofibroblasts were isolated and cultured with G-CSF. Collagen, MMP-9 and MMP-13 expression by myofibroblasts was examined. The BM-mismatched mice model was used to examine the change of BM-derived myofibroblasts and non-myofibroblastic BM cells by G-CSF in the pancreas.

**Results:**

The pancreatic collagen expression were significantly decreased in the G-CSF-treated group sacrificed at week 9. While collagen produced from myofibroblasts was not affected by G-CSF, the increase of MMP13 expression was observed *in* *vitro*. There were no effect of G-CSF in the number of myofibroblasts and BM-derived myofibroblasts. However, the number of non-myofibroblastic BM cells and macrophages were significantly increased in the pancreata of cerulein- and G-CSF-treated mice, suggesting a potential anti-fibrotic role of non-myofibroblastic BM cells and macrophages stimulated by G-CSF.

**Conclusions:**

Our data indicated that G-CSF contributed to the regression of pancreatic fibrosis. The anti-fibrotic effects were possibly through the stimulation of MMP-13 from myofibroblasts, and the enhanced accumulation of non-myofibroblastic BM cells and macrophages in the pancreas.

## Introduction

Chronic pancreatitis (CP) is a disease characterized by inflammation, parenchymal atrophy, and extensive fibrosis [1]. The fibrosis often results from repeated episodes of acute inflammation and leads to the permanent destruction of the pancreas and the loss of acinar and islets cells [2]. Clinically most patients suffer from chronic abdominal pain and symptoms of exocrine and endocrine insufficiencies such as malabsorption, diarrhea and diabetes despite therapy. Although the process can take many years [3], to date, there are no effective methods to stop fibrosis and progression.

Bone marrow (BM)-derived cells have been shown to participate in the process of inflammation and fibrosis. During inflammation, BM cells migrate into the diseased organ and further differentiate into myofibroblasts. These BM-derived myofibroblasts has been showed to participate in fibrosis in organs including heart, lung, liver and kidney [4]–[7]. Conversely, there is a growing body of evidence that the BM-derived cells may contribute to the regression of fibrosis [8]. It has been shown that the BM-derived mesenchymal stem cells can reduce the fibrosis in the injured lung [9]. The protective effects are associated with the migration of BM-derived cells, the increased circulating level of granulocyte colony-stimulating factor (G-CSF), and the decrease of inflammatory cytokines. In a mouse model of liver fibrosis, the BM-derived cells express matrix metalloproteinase (MMP)-13 and MMP-9 and contribute to the regression of liver fibrosis [5]. In pancreas, it has been demonstrated that BM-derived cells contribute to the pancreatic myofibroblasts population and be involved in the process of pancreatic fibrosis [10]–[13]. However, whether BM-derived cells can ameliorate the degree of fibrosis in the pancreas is still unclear.

G-CSF causes the mobilization of BM stem cells into the peripheral blood and has been applied to all types of leukopenia clinically [14], [15]. The anti-fibrotic effects of G-CSF have been tested in many animal models and human clinical trials and the results are promising. In a swine model of myocardial infarction, the administration of G-CSF accelerates angiogenesis and reduces fibrosis [6]. In a mouse model of liver fibrosis, the administrating of G-CSF not only enhances BM-derived cells migration to fibrotic tissues, but also accelerates the regression of fibrosis [5]. Furthermore, G-CSF can also enhance the proliferation of hepatic progenitor cells in both rodents and humans [16], [17]. In the acute pancreatitis animal models, G-CSF shows a protective effects through promoting the migration of BM-derived cells, increasing the number of neutrophils, improving expression of opsonin receptors on neutrophils and enhancing their functions against bacteria [18]–[20]. However, whether G-CSF plays an anti-fibrotic role in CP is still unknown.

In the present study, we aim to study the potential anti-fibrotic role of G-CSF. CP was induced in mice by repeated injection of cerulein. Mice with CP were further treated with G-CSF or normal saline (NS) and sacrificed at different time points. Pancreatic histology, pancreatic matrix metallopeptidase 9 (MMP-9), MMP-13 and collagen expression were examined to evaluate its effects on CP. Furthermore, pancreatic myofibroblasts were isolated and cultured with G-CSF to test its effect on collagen, MMP-9 and MMP-13 production from myofibroblasts. To further evaluate G-CSF effects on BM cell mobilization and engraftment in CP, BM from transgenic male mice that constitutively express green fluorescent protein (GFP) was transplanted into wild type (WT) female mice. CP was induced in these BM-transplanted animals and further treated with G-CSF or NS. The myofibroblasts, BM-derived myofibroblasts and non-myofibroblastic BM cells including macrophages, T cells and B cells were examined. The results indicated that the degree of fibrosis and collagen I (ColI) in the pancreatic tissues were decreased in G-CSF-treated mice sacrificed at week 9. While no direct effect of G-CSF on collagen production of myofibroblasts, MMP-13 was increased from myofibroblasts treated with G-CSF. Although the number of myofibroblasts and BM-derived myofibroblasts were not changed by G-CSF, the enhanced infiltration of BM derived cells and the increased number of macrophages were observed in the pancreata of cerulein- and G-CSF-treated mice. These results suggest that G-CSF may play an anti-fibrotic role in CP through both the increase of MMP-13 production from myofibroblasts and the enhanced accumulation of macrophages stimulated by G-CSF.

## Materials and Methods

### Experimental protocols

**Figure 1:**
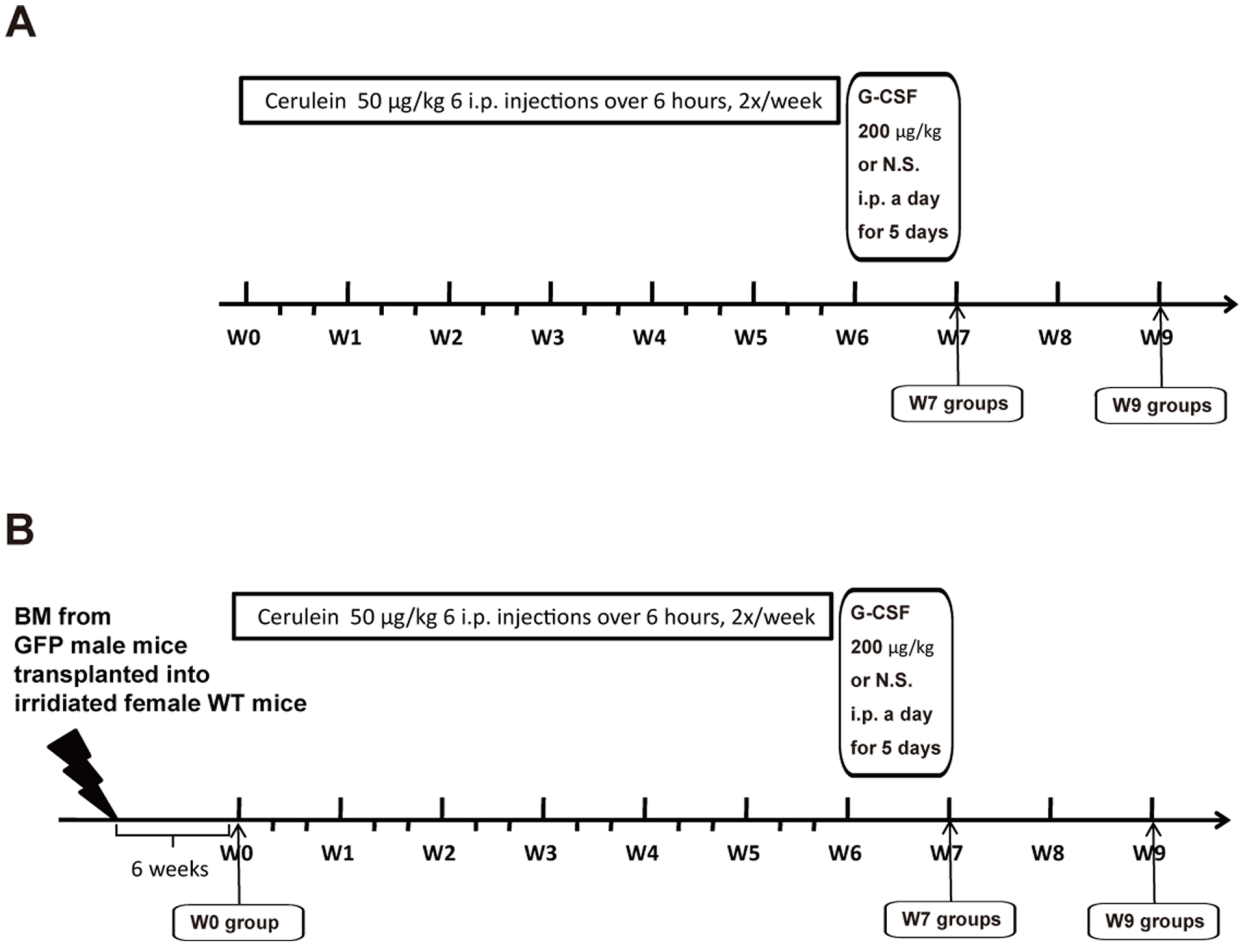
Experimental designs. (A) CP was induced by administration of 6 i.p. injection of cerulein (50 ug/kg) at 1-h interval, 2 days per week, for a total of 6 weeks. Control female mice received NS in a similar manner. G-CSF (200 ug/kg/day) or NS was then i.p injected once a day for 5 consecutive days. Groups of mice were sacrificed at 7 (W7 groups) and 9 (W9 groups) weeks after the beginning of cerulein treatment, and the pancreases were removed and processed for further analysis. (B) Sex mismatched BM transplantation was performed using 8-week-old WT C57BL/6 female mice as recipients and 8-week-old male GFP mice as donors after 5 Gy whole body gamma irradiation to ablate their BM. Six weeks after transplantation, Mice (W0 group) were sacrificed to determine the success of re-established haematopoietic system by transplanted male GFP BM. The mice were then treated in the same experimental design mentioned in A.

The procedures for animal experiments were performed under U.S. National Institutes of Health guidelines, and the Chang Gung Institutional Animal Care and Use Committee Guide for Care and Use of Laboratory Animals (IACUC Approval NO 2011081601). The first experiment aimed to test the anti-fibrotic effects of G-CSF and the protocol was shown in Fig. 1A. Twenty-four wild-type C57BL/6 female mice were assigned to one of four treatments shown in S1 Table: (1) Control mice were treated with NS 50 µl intraperitoneally (i.p.) 6 times a day, two days a week for 6 consecutive weeks and N.S. 50 µl i.p. for 5 consecutive days on week 7; (2) Cerulein mice were treated with cerulein at 50 µg/kg body weight 6 times a day, two days a week for consecutive 6 weeks and NS 50 µl i.p. for consecutive 5 days on week 7; (3) G-CSF mice were treated with NS 50 µl i.p. 6 times a day, two days a week for consecutive 6 weeks and G-CSF 200 ug/kg/day i.p. for consecutive 5 days on week 7; (4) Cerulein and G-CSF mice were treated with cerulein at 50 µg/kg body weight 6 times a day, two days a week for 6 consecutive weeks and then G-CSF 200 ug/kg/day i.p. for 5 consecutive days on week 7. Blood and pancreatic tissues were harvested at 7 weeks (W7 group) or 9 weeks (W9 group) after first cerulein or NS injection. The second experiment aimed to test the effects of G-CSF on BM-derived cells and protocol is shown in Fig. 1B. To create BM-mismatched mice, eight week-old WT C57/BL6 female recipient mice (National Laboratory Animal Center, Taipei, TW) underwent whole body gamma irradiation with 5 Gy to ablate their BM, followed immediately by tail vein injection of whole male GFP BM cells (2×10^6^), resuspended in 0.1 ml sterile phosphate-buffered saline (PBS) with 2% foetal calf serum (FCS). Six weeks after the BM transplantation, three mice (W0 group) were sacrifice to determine the success of re-established haematopoietic system by transplanted male GFP BM. Then 50 BM-transplanted female mice were assigned to one of four treatments as mentioned above and shown in S2 Table.

### Determination of amylase and lipase

The blood samples were centrifuged at 2000 rpm for 10 minutes to separate the serum. The activities of amylase in the serum were determined using AMYL and LIPA microslide (Vitros, Rochester, NY, USA) by Ektachem DT System.

### Hydroxyproline assay

The level of hydroxyproline was examined by using Biovision hydroxyproline assay kit (Biovision, Milpitas, CA, USA). Briefly, the pancreas tissues were homogenized in dH_2_O and then hydrolyzed in 12 N HCl, at 120°C for 3 hours. The lysates were separated by centrifuging at 10000 × g for 3 minutes. Then 10 ul of hydrolyzed samples were transferred to a 96-well plate and placed in a 60°C oven. After the samples were dried, 100 ul the Chloramine T/Oxidation buffer were added to each sample well and incubated for 5 minutes at room temperature. After that, 100 ul dimethylaminobenzaldehyde reagent was added to each well and incubated for 90 minutes at 60°C. Finally, the absorbance at 560 nm of wells was measured in a micro-plate reader.

### Collagen staining

Picro-Sirius red (VWR International) was used for staining intrapancreatic collagen. Quantitative analysis of collagen deposition was performed by digitized image analysis with NIH-ImageJ software [21]. The total pancreatic tissue area was distinguished from the background according to a difference in light density. The total amount of collagen (stained in red) was measured and expressed as a percentage of the total pancreatic surface.

### Protein extraction and Western blot assay

The freshly harvested pancreatic tissues were homogenized and lysed in ice-cold RIPA lysis buffer (150 mM NaCl, 1.0% NP-40, 0.5% sodium deoxycholate, 0.1% sodium dodecyl sulfate, 50 mM Tris [pH7.5], 1 mM PMSF, 10 g/ml Leupeptin) for 60 minutes by vortexing every 5 minutes. The lysates were separated by centrifugation at 14,000×g for 10 minutes at 4°C. The supernatants were collected, aliquoted, and stored at −80°C until Western blot assay conducted. 20 µg of total protein per lane was separated by 10% SDS-PAGE gel and then were transferred to a polyvinylidene fluoride membrane. The membrane was blocked by 5% FBS in 1X Tris-Buffered Saline with 0.1% Tween-20 and then incubated with anti-ColI antibody (dilution 1∶10000, Abcam, Cambridge, MA, USA), MMP-9 antibody (1∶5000 Abcam), MMP-13 antibody (1∶10000, Abcam) and GAPDH antibody (1∶15000, Proteintech, Chicago, IL, USA) at room temperature for 90 minutes. The membrane was then incubated with diluted horseradish peroxidase-linked anti-rabbit IgG (1∶1000, Cell Signaling Technology, Boston, MA, USA) for 1 hour at room temperature. The membranes were washed for 5 minutes by 3 times between each step. The protein-antibody complex was detected by the chemiluminescent substrate (Cell Signaling), the emitted light was captured on an X-ray film, and the intensities of bands were semi-quantified by ImageJ software.

### Isolation and culture of pancreatic activated myofibroblasts with or without G-CSF

Pancreatic activated myofibroblasts were isolated by the method described by Saotome et al. [22]. The pancreatic tissue was evaluated to be normal by light microscopic observation. Resected pancreatic tissue was cut into small pieces in HBSS using a sterile razor blade. HBSS containing 3 U/ml dipase, type IV collagenase and 10 µg/ml deoxyribonuclease was injected into the specimens using a 27-G needle, and the specimens were shaken for 30 minutes at 37°C. After centrifugation for 5 minutes at 400 rpm, the supernatant was washed twice with dulbecco’s modified eagle medium (DMEM) medium containing 10% FBS and then the precipitate with the 5 ml if same medium was seeded into culture dishes. The isolated cells were cultured at 37°C in 8% CO_2_ and the culture medium was changed every 2 days. After reaching confluence, the monolayers of cells were trypsinized and passaged. All studies were performed on passage three to five. The purity of myofibroblasts was assessed by cytoplasmic staining for α-SMA. To test the effects of G-CSF on myofibroblasts *in* *vitro*, 4×10^5^ α-SMA(+) cells were seeded into 10 cm plates, and cultured in DMEM with 10% FCS at 37°C overnight. Cells were further cultured in the DMEM medium with or without 100 ng/ml G-CSF for 24 hr. Then cells were harvested and the total proteins were extracted for hydroxyproline assay and Western blot analysis.

### FACS analysis of peripheral blood

Peripheral blood was analyzed for GFP expression using fluorescence-activated cell sorting (FACS) to confirm the re-established haematopoietic system by transplanted male GFP BM. Terminal blood samples (0.7 ml) were taken by cardiac puncture into heparin-coated tubes. A volume of 0.2 ml of blood was aliquoted into separate 15 ml Falcon tubes. Next, 1.8 ml of lysing solution (Becton-Dickinson, Bedford, USA) was added to each tube, vortexed and left to incubate for 3 minutes in the dark. After incubation, 13 ml of PBS were added to each tube to dilute the lysing solution and centrifuged at 400 × g for 4 minutes. The resultant supernatant was removed and the pellet was resuspended in cold PBS on ice. The cells were analyzed by a FACS Aria (Becton Dickinson Biosciences) following the instrument configuration and sorting procedure recommended by the manufacturer. Each analysis included at least 10 000 events.

### Immunohistochemistry

Sections cut at 4 µm were dewaxed, incubated in 1.8% v/v hydrogen peroxide in methanol for 15 min to block endogenous peroxidases, then taken through graded alcohols to PBS. The sections were then incubated in 20% v/v acetic acid in methanol to block endogenous alkaline phosphatases. Antigen retrieval treatment was performed by microwaving (700 W) sections in BD Retrievagen A solution (550524, BD Pharmagen) for 10 minutes. To reduce non-specific background staining, sections were next pre-incubated with 1% bovine serum albumin (A4503, Sigma-Aldrich) for 30 minutes. The first antibodies included monoclonal anti-α-smooth muscle actin (α-SMA; A-2547, Sigma-Aldrich) at a 1/5000 dilution, monoclonal anti-F4/80 (ab6640, Abcam) at a 1/100 dilution, monoclonal anti-CD45R/B220 antibody (550286; BD Pharmingen) at a 1/200 dilution and monoclonal anti-CD3 antibody (ab16669, Abcam) at a 1/100 dilution. The secondary antibodies were used including a biotinylated polyclonal rabbit anti-mouse immunoglobulin at a dilution of 1∶300 (E0464, Dako), anti-rat IgG biotinylated antibody (AK 5004, Vectastain) diluted at 1∶200 or anti-rabbit IgG biotinylated antibody (ab64256, Abcam) at a dilution of 1∶200. Tertiary layer of peroxidase-conjugated streptavidin (P0397; Dako) was used at a dilution of 1∶500. Slides were developed in DAB and counterstained lightly with haematoxylin, dehydrated and mounted in DPX-type mount.

### Immunofluorescence

The 5 µm frozen section was blocked with with 1% bovine serum albumin (A4503, Sigma-Aldrich) for 30 minutes. Then monoclonal anti-mouse α-SMA (A-2547, Sigma-Aldrich) was used at a dilution of 1∶5000 as primary antibody for 1 hour. The goat anti-mouse Rhodamine TRITC (M1061; Leinco Technologies) was used as secondary antibody with a dilution of 1∶500 for 1 hour at room temperature. Between each step, the slides were washed in PBS buffer 5 minutes for 3 times. The slides were rinsed then counterstained and mounted in Vectashield hard set mounting medium with DAPI (H-1500; Vector Laboratories).

### Cell counting

For each mouse pancreas, sections were analyzed by digitally photographing 10 consecutive microscope fields at x400 total magnification. The number of myofibroblasts (α-SMA positive), BM cells (GFP positive), macrophages (F4/80 positive) were quantified and the proportion of BM-derived (both α-SMA and GFP positive) myofibroblasts was expressed as a percentage.

### Statistics

Results are expressed as mean ± standard error of the mean (SEM). Mean values of the experimental groups were compared by Student’s unpaired two tail t-tests. *P* values of less than 0.05 were considered to be statistically significant.

## Results

### G-CSF did not cause ongoing acinar damage but reduced collagen deposition

**Figure 2:**
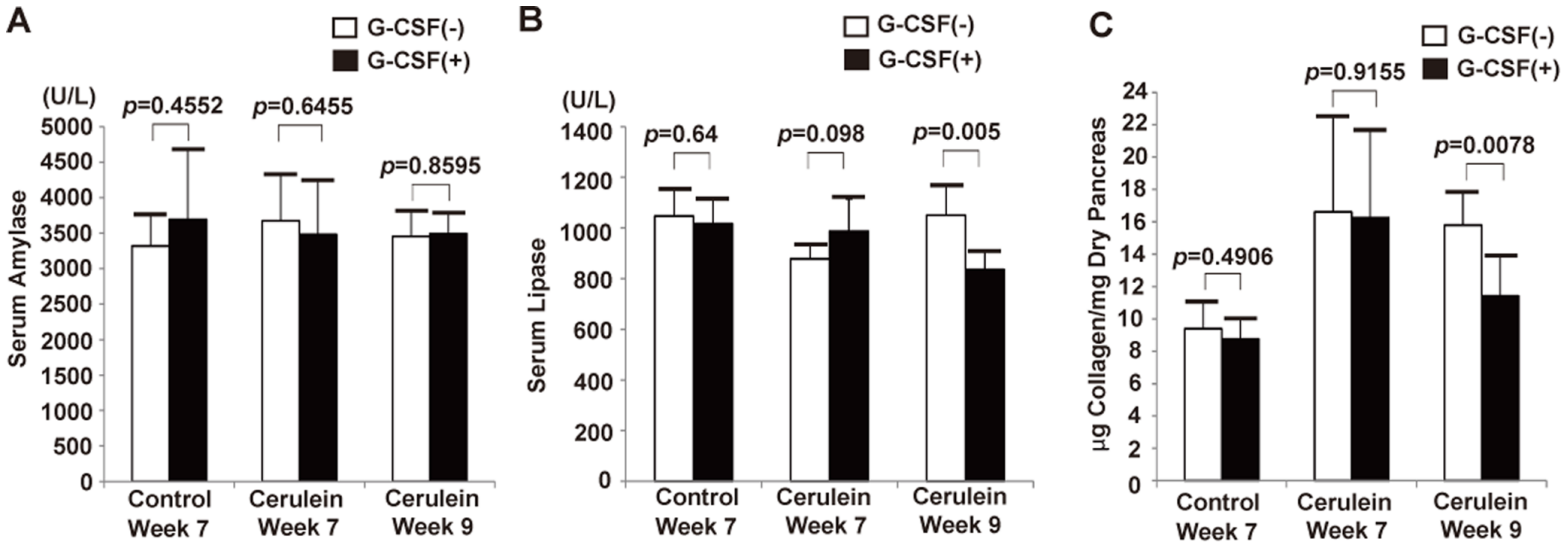
The serum amylase, lipase and pancreatic collagen in experimental mice. (A) Serum amylase level. The serum amylase level was not significantly changed by G-CSF treatment in either control mice or cerulein-treated mice. Values are mean ± SD (n = 3–6). (B) Serum lipase level. The serum lipase level was significantly decreased in cerulein- and G-CSF-treated mice at week 9. Values are mean ± SD (n = 3–6). (C) Pancreatic collagen measurement by hydroxyproline assay. The collagen amount in pancreas was not changed by G-CSF treatment in either control mice or cerulein-treated mice sacrificed at week 7. The collagen amount was significantly decreased in the pancreas of cerulein- and G-CSF-treated mice compared with cerulein-treated mice sacrificed at week 9. Values are mean ± SD (n = 3–6).

**Figure 3:**
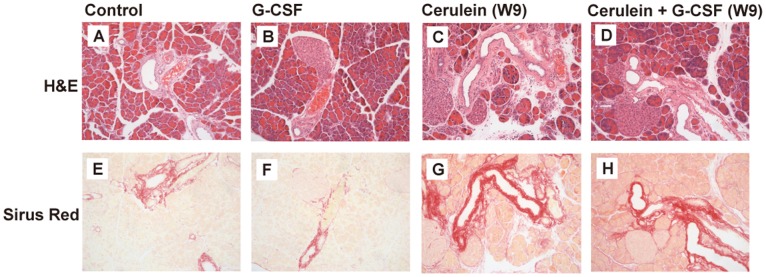
Histology of pancreas. The staining show The H&E and Sirius Red staining in the pancreas from control (A, E) and G-CSF (B, F) groups showed normal pancreatic structure and collagen distribution. Collagen deposition, acinar cell atrophy, inflammation and structure changes in the pancreas were demonstrated in the cerulein group (C, G) and cerulein+G-CSF group sacrificed at week 9 (D, H).

The elevation of serum amylase and lipase has been used as markers for acute pancreatitis and ongoing acinar damage. The serum amylase and lipase were measured to detect acute inflammation and pancreatic acinar damage. The results showed there were no differences of serum amylase between G-CSF-treated and non-G-CSF-treated mice in control group and cerulein-treated groups (Fig. 2A). Furthermore, a modest decrease of serum lipase level was observed in cerulein- and G-CSF-treated mice at week 9 (Fig. 2B). These findings suggested that no acute inflammation and acinar damage was continuing after the discontinuance of cerulein with or without G-CSF administration. Hematoxylin-eosin and Sirius Red stained slides also revealed normal pancreatic structure and collagen distribution in control mice either with or without G-CSF treatment (Fig. 3A, B, E and F). However, the cerulein-treated mice with or without G-CSF exhibited CP and fibrosis including acinar loss, stromal fibrosis, and inflammatory cell infiltration (Fig. 3C, D, G and H).

To test whether the degree of collagen deposition was changed by G-CSF, the level of collagen in the pancreas was measured by hydroxyproline assay. The results showed that the level of collagen in pancreata was elevated significantly by repeated cerulein treatment (Fig. 2B). However, a significant decrease of collagen level was observed in the cerulein- and G-CSF-treated mice sacrificed at week 9 compared with cerulien-treated mice without G-CSF (15.8±2 vs 11.4±2.5 µg collagen/mg dry pancreas, p = 0.0078, Fig. 2C). This suggested that collagen deposition was reduced by G-CSF administration.

**Figure 4:**
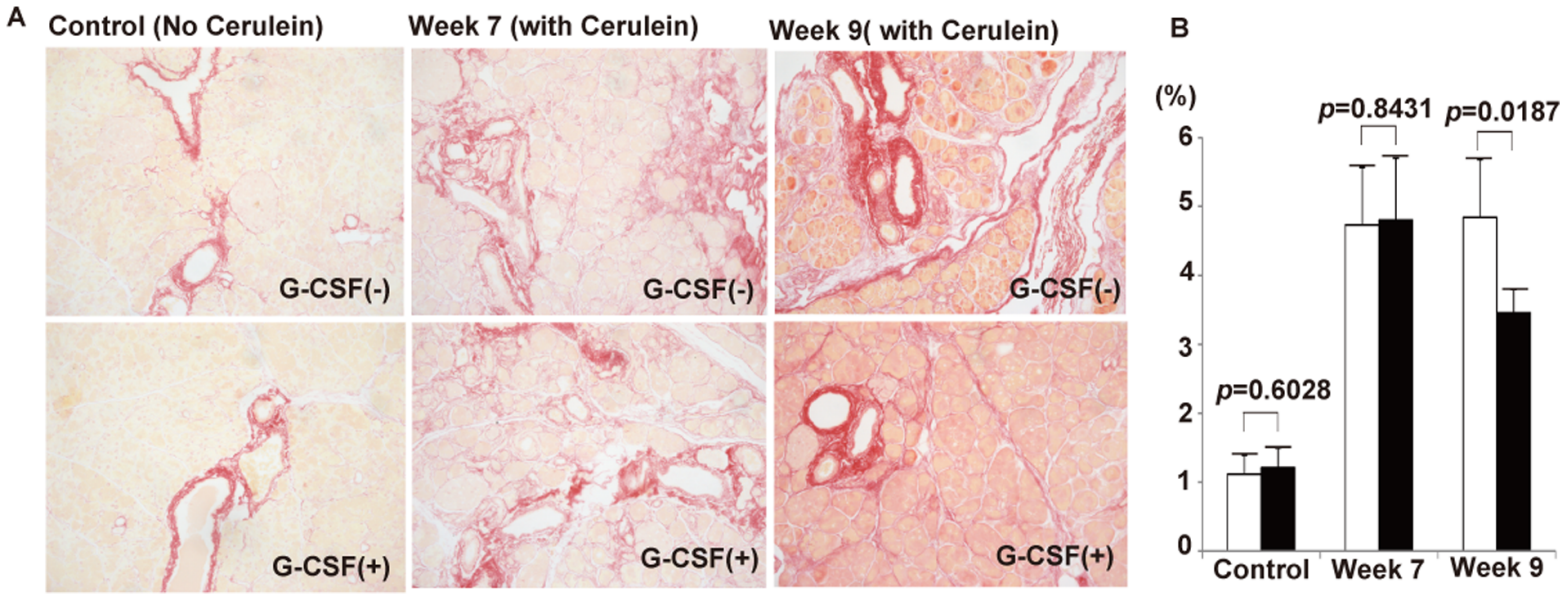
Semi-quantification of pancreatic fibrosis by Sirius Red staining. (A) The Sirius Red staining of pancreas from control mice and cerulein-treated mice with or without G-CSF sacrificed at week 7 and week 9. (B) The degree of pancreatic fibrosis was semi-quantified by measuring the relative areas of fibrosis using ImageJ software. Values are mean ± SD (n = 3–6). The result reveals that the degree of pancreatic fibrosis is significantly decreased in cerulein- and G-CSF treated mice sacrificed at week 9.

**Figure 5:**
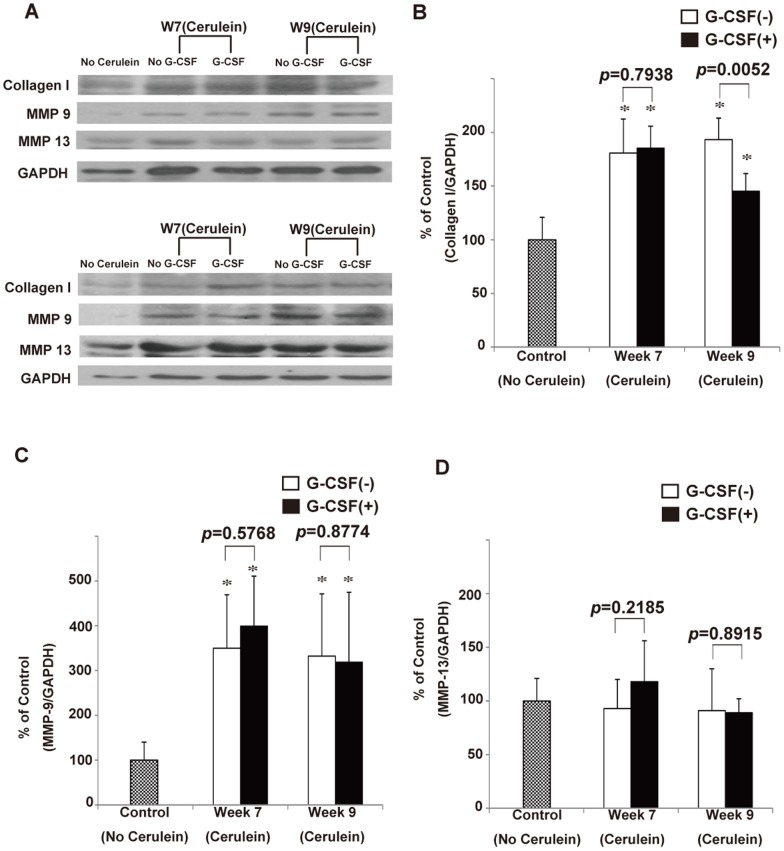
Semi-quantification of pancreatic collagen I, MMP-9 and MMP-13 by Western blot analysis. (A) The Western blot analysis of collagen I, MMP-9 and MMP-13 expression in the pancreas of control mice and cerulein-treated mice with or without G-CSF sacrificed at week 7 and week 9. (B) The relative amount of pancreatic collagen I. Values are mean ± SD (n = 3–6). This result reveals that the relative amount of collagen I is significantly decreased in cerulein- and G-CSF treated mice sacrificed at week 9. (C) The relative amount of pancreatic MMP-9. Values are mean ± SD (n = 3–6). This result reveals that no significant change of MMP-9 was caused by G-CSF treatment in cerulein-treated mice sacrificed at week 7 and week 9. (D) The relative amount of pancreatic MMP-13. Values are mean ± SD (n = 3–6). This result reveals that no significant change of MMP-13 was caused by G-CSF treatment in cerulein-treated mice sacrificed at week 7 and week 9.

To confirm the above finding, we further quantified the degree of pancreatic fibrosis between G-CSF-treated and non G-CSF-treated cerulein-treated mice by calculating the area of positive collagen deposition. The results showed the area of fibrosis was similar between G-CSF-treated and non G-CSF-treated cerulein-treated mice sacrificed at week 7 (4.83±0.91 vs 4.74±0.85%; *p* = 0.8431), while there was a significant difference between G-CSF-treated and non G-CSF-treated mice with CP sacrificed at week 9 (3.52±0.34 vs 4.86±0.84%; *p* = 0.0187; Fig. 4B). This result again suggested that G-CSF promote the regression of fibrosis. Moreover, the ColI Western blot analysis showed the relative amount of ColI was different between G-CSF-treated and non G-CSF-treated mice with CP sacrificed at week 9 (1.452±0.164 vs 1.933±0.201; p = 0.0052), but no difference was observed at week 7 (1.853±0.204 vs 1.808±0.317; p = 0.7938; Fig. 5A and B). Taken together, our results suggested that G-CSF treatment cannot change the level of collagen and the degree of pancreatic fibrosis immediately within 3 days, but can ameliorate them gradually 2 weeks later.

### G-CSF did not change the level of MMP-9 and MMP-13 in the pancreas

It has been suggested that MMP-9 and MMP-13 contribute to the regression of organ fibrosis. The Western blot analysis of MMP-9 and MMP-13 were performed to evaluate whether G-CSF can increase the expression of anti-fibrotic enzymes in mice with CP. The results showed that the level of MMP-9 was significant increased in cerulein-treated mice compared with control mice (Fig. 5A and C). On the contrary, the level of MMP-13 was not changed by cerulein injection (Fig. 5A and D). The level of MMP-9 and MMP-13 seems to be slightly increased by G-CSF in cerulein-treated mice at week 7, however, both increases were not significant. The administration of G-CSF did not significantly change the level of MMP-9 and MMP-13 of pancreata from mice with CP at week 9 (Fig. 5A, C and D), indicating the anti-fibrotic MMP-9 and MMP-13 were not stimulated in the pancreata significantly by G-CSF administration.

### G-CSF did not increase collagen production but did modestly stimulate the MMP-13 production from myofibroblasts in vitro

**Figure 6:**
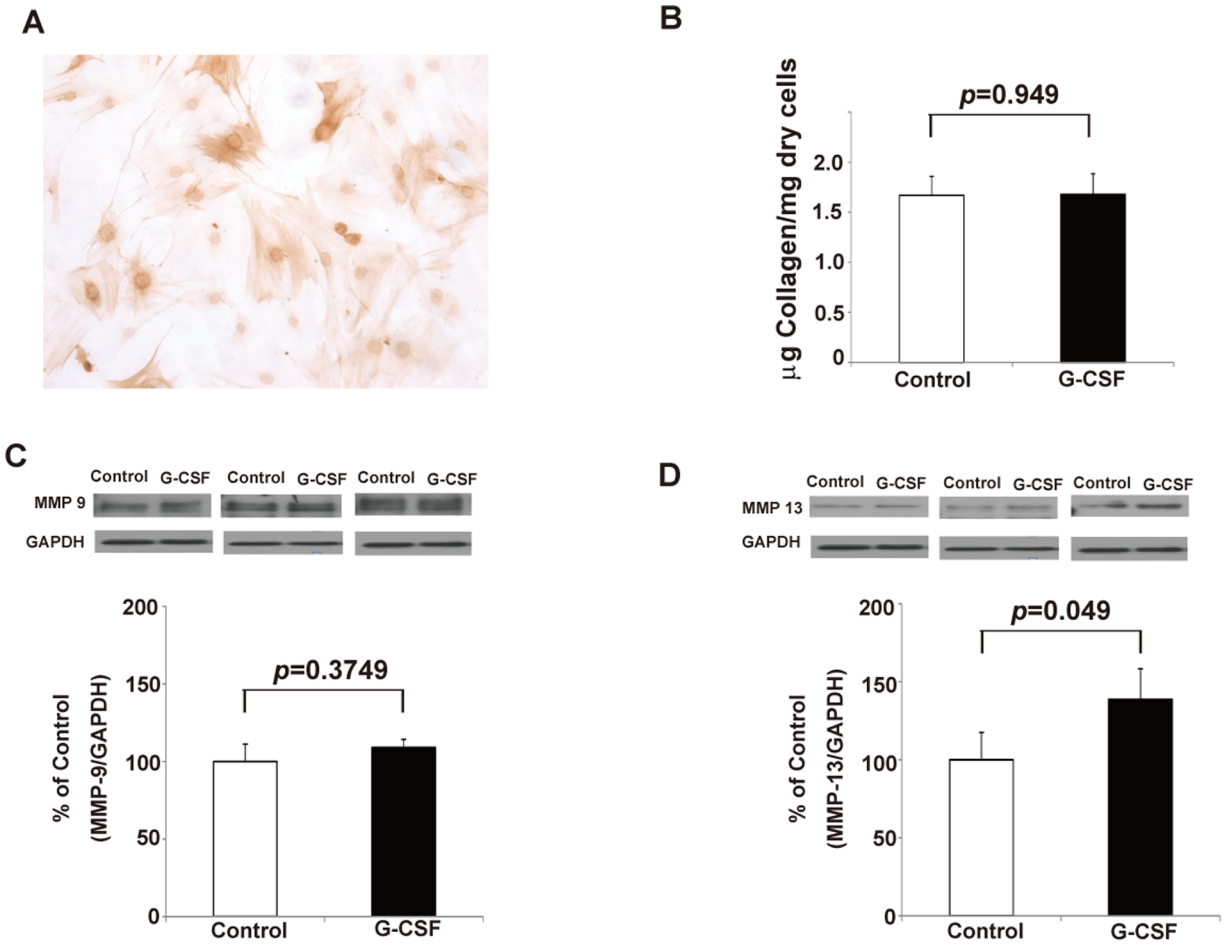
The effects of G-CSF on isolated pancreatic myofibroblasts *in* *vitro*. (A) The α-SMA positive myofibroblasts isolated from pancreas of control mice. (B) Myofibroblast collagen measurement by hydroxyproline assay. Collagen expression by myofibroblasts *in* *vitro* was not changed by G-CSF (n = 3). (C) The Western blot analysis and relative amount of MMP-9 from myofibroblasts. Values are mean ± SD (n = 3). This result reveals that the production of MMP-9 from myofibrolbasts was not changed by G-CSF *in* *vitro*. (D) The Western blot analysis and relative amount of MMP-13 from myofibroblasts. Values are mean ± SD (n = 3). This result reveals that the production of MMP-13 from myofibrolbasts was modestly and significantly increased by G-CSF *in* *vitro*.

To evaluate whether G-CSF has direct effects on pancreatic myofibroblasts, the isolated myofibroblasts from control mice were cultured with G-CSF. The isolated myofibroblasts were confirmed by a-SMA staining (Fig. 6A). The amount of collagen was evaluated by hydroxyproline assay, and levels of MMP-9 and MMP-13 were examined by Western blot. The results showed that the amount of collagen and the level of MMP-9 from myofibroblasts were not increased by G-CSF (Fig. 6B and C). On the contrary, the production of MMP-13 from myofibroblasts cultured with G-CSF was modestly but significantly increased compared with control (1.387±0.197 vs 1±0.175, p = 0.049, Fig. 6D), indicating that anti-fibrotic effects of G-CSF may be partially through the increase of MMP-13 production from myofibroblasts.

### G-CSF did not change the number of myofibroblasts

**Figure 7:**
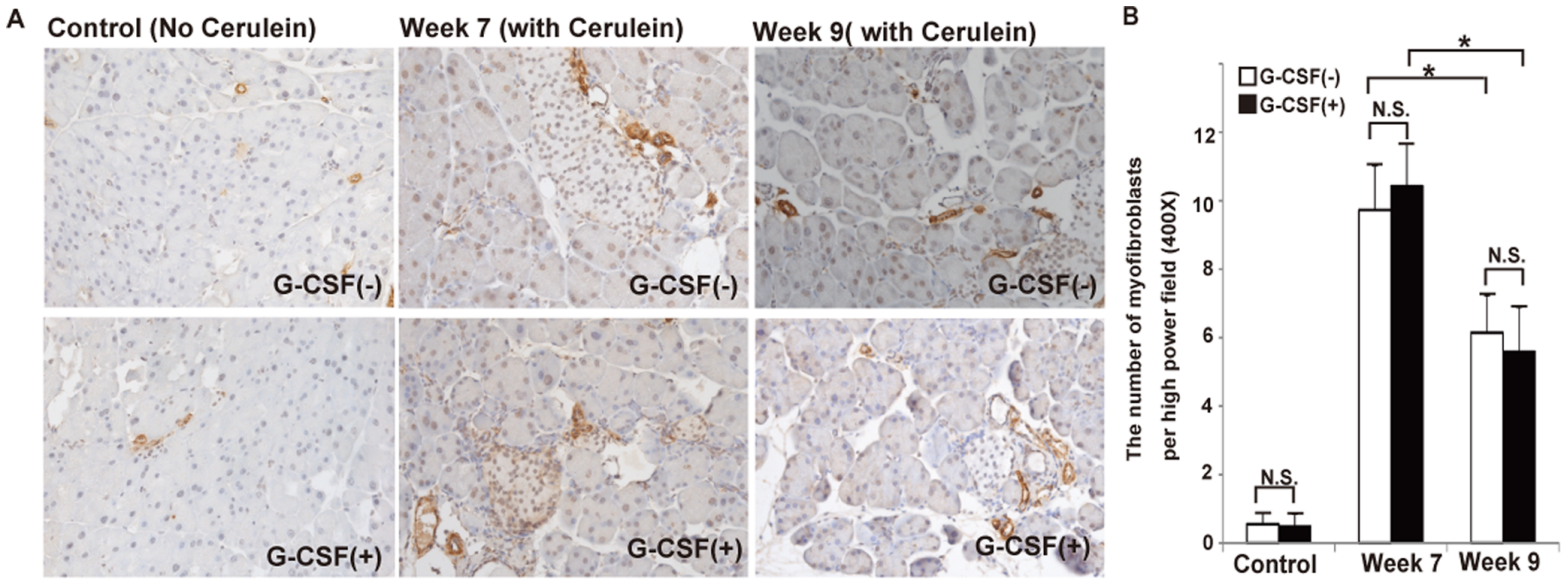
The effects of G-CSF on pancreatic myofibroblasts *in* *vivo*. (A) The IHC staining of α-SMA from cerulein-treated mice with or without G-CSF sacrificed at week 7 and week 9. (B) The number of α-SMA(+) cells was counted and analyzed statistically by randomly selecting 10 fields per section. Values are mean ± SD (n = 3–6). NS, not significant.* P<0.05. This result reveals that the number of α-SMA(+) cells was significantly decreased at week 9 compared with week 7. There were no significant differences of α-SMA(+) cells between G-CSF- and non G-CSF-treated mice at week 7 and week 9.

The activated myofibroblasts play a crucial role in pancreatic fibrosis. To evaluate the effects of G-CSF on activated myofibroblasts, the α-SMA positive cells between cerulein-treated mice with and without G-CSF were stained and semi-quantified (Fig. 7). The number of myofibroblasts was significantly decreased in pancreatic tissues from cerulein-treated mice sacrificed at week 9 compared with those at week 7 (6.15±1.08 vs 9.725±1.298/HPF; p = 0.011). The decrease of myofibroblasts between week 7 and week 9 was also observed in cerulein- and G-CSF-treated mice (5.6±1.28 vs 10.425±1.239/HPF; p = 0.003). However, the G-CSF did not change the number of myofibroblasts at week 7 (10.425±1.239 vs 9.725±1.298/HPF; p = 0.5247) and at week 9 (5.6±1.283 vs 6.15±1.081/HPF; p = 0.5911), suggesting the number of myofibroblasts in the pancreata was not changed by G-CSF administration.

### Hematological reconstitution in the recipient mice

To evaluate the effects of G-CSF on BM-derived cells, the BM from GFP (+) transgenic mice was transplanted into wide type female mice. First, the hematological reconstitution after irradiation and BM transplantation was evaluated. WT mice, GFP transgenic mice and recipient mice were sacrificed 6 weeks after BM transplantation and the peripheral bloods were sent for FACS analysis. The results showed that the GFP positive donor cells constituted 46.2±1.42% of peripheral hematopoietic cells from recipient mice, while in the GFP transgenic mice, 86.5±1.19% of peripheral cells displayed GFP fluorescence by FACS (S1 Fig.), indicating that BM of recipient mice were partially reconstituted by GFP positive BM cells from donor.

### G-CSF enhanced the migration of BM cells but did not increase in the number of BM-derived myofibroblasts

**Figure 8:**
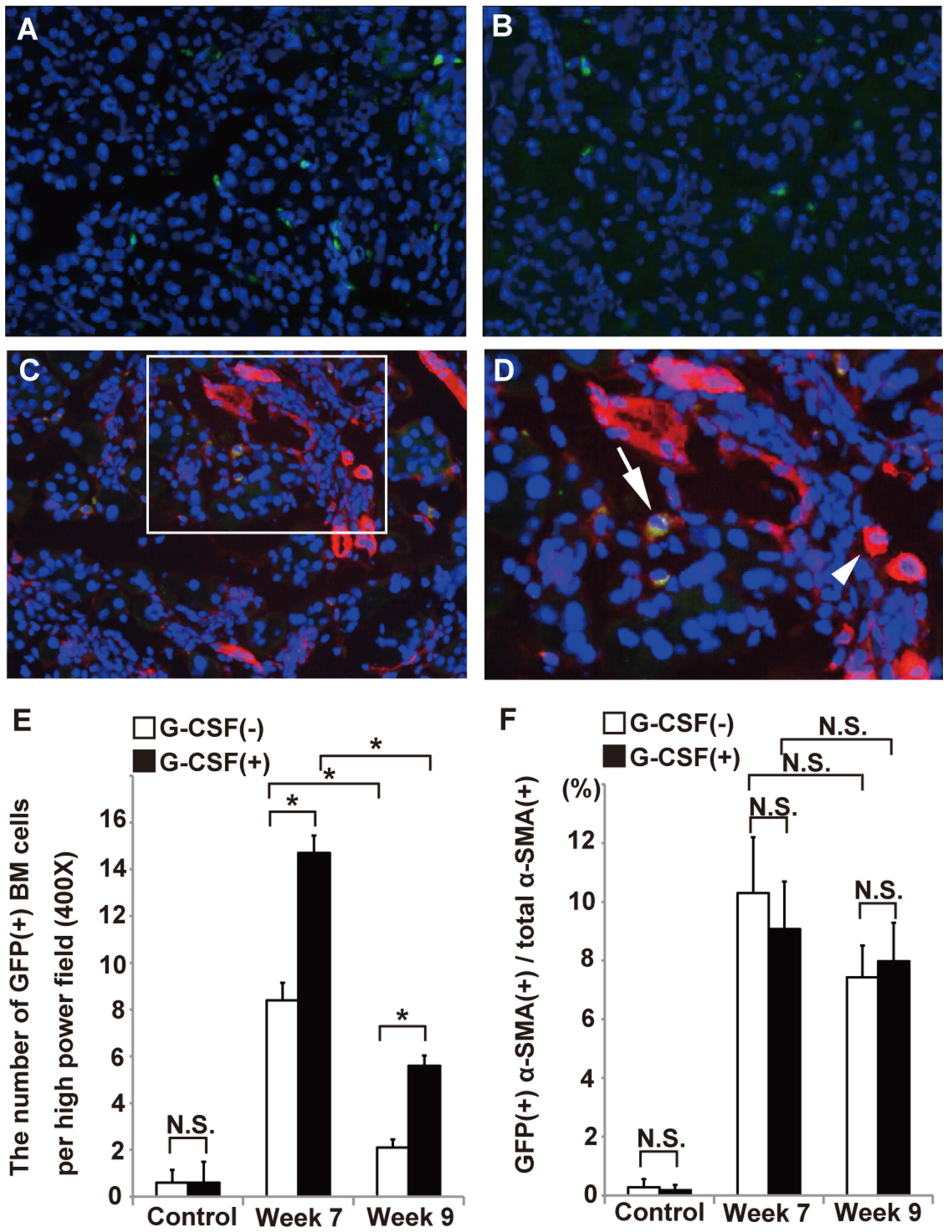
The effects of G-CSF on BM cells and BM-derived myofibroblasts *in* *vivo*. The GFP(+) cells in pancreatic tissues from cerulein-treated mice with G-CSF (A) and without G-CSF (B). The GFP(+) cells were stained with α-SMA (C, D). Arrow indicates GFP(+) cells co-expressing α-SMA. Arrow head indicates α-SMA(+) cell. (E) The number of GFP(+) cells in the pancreas. (F) The proportions of GFP(+)α-SMA(+) cells/total α-SMA(+) cells (excluding vascular cells) were examined in the pancreatic tissues removed at week 7 and week 9. Values are mean ± SEM (n = 9). NS, not significant.* P<0.05.

The number of GFP-positive BM cells in the pancreata of cerulein-treated mice at week 9 was approximately one quarter the number observed at week 7 (2.1±0.30 vs 8.4±0.65/HPF; p = 0.00007). Likewise, in the cerulein and G-CSF treated mice, there was a very significant decline in GFP-positive BM cells from week 7 to week 9 (14.7±0.64 vs 5.6±0.37/HPF; p = 0.000006). Furthermore, compared with cerulein-treated mice, the administration of G-CSF into cerulein-treated mice significantly increased the number of GFP positive BM cells migrating into pancreas from mice sacrificed at week 7 (8.4±0.65 vs 14.7±0.64/HPF; p = 0.00002) and week 9 (2.1±0.30 vs 5.6±0.37/HPF; p = 0.00002), respectively (Fig. 8A, B and E). These results indicated the G-CSF not only reduced the rate of BM cells decline, but also enhanced the migration of BM cells in the fibrotic pancreatic tissues.

It has been suggested that BM-derived myofibroblasts contribute to organ fibrosis. To evaluate the effects of G-CSF on BM-derived myofibroblasts, the immuno-fluorescent α-SMA staining was applied on frozen tissues and the percentage of GFP and α-SMA double positive cells in the total α-SMA positive cells was semi-quantified (Fig. 8C, D and F). The results showed that the percentage of BM-derived myofibroblasts in total myofibroblasts was not changed by G-CSF on week 7 (9.07±1.39 vs 10.29±1.65%; p = 0.36) and week 9 (7.98±1.13 vs 7.43±0.94%; p = 0.42) respectively, suggesting G-CSF did not enhance the transdifferentiation of BM cells into myofibroblasts.

### G-CSF stimulated macrophage infiltration

**Figure 9:**
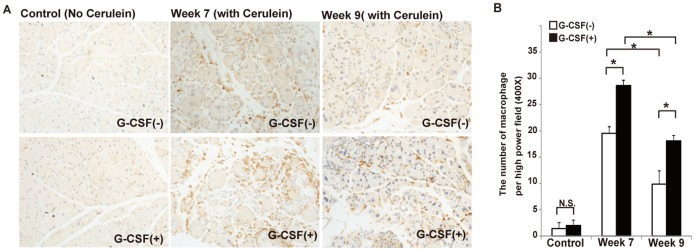
The effects of G-CSF on macrophages within pancreas *in* *vivo*. (A) The IHC staining of F4/80 from cerulein-treated mice with or without G-CSF sacrificed at week 7 and week 9. (B) The number of F4/80(+) cells was counted and analyzed statistically by randomly selecting 10 fields per section. Values are mean ± SD (n = 3–6). NS, not significant.* P<0.05. This result reveals that the number of F4/80(+) macrophage in the pancreas was significantly increased in cerulein- and G-CSF-treated mice at week 7 and week 9 compared with cerulein-treated mice.

Because an increased number of non-myofibroblastic BM cells were observed in pancreata from cerulein- and G-CSF-treated mice, we further analyzed three populations of hematopoietic BM cells involved in inflammation including macrophages, T cells and B cells by IHC staining. The results showed that there were not a major infiltration of T cells and B cells in cerulein-treated mice with or without G-CSF (S2 and S3 Figs.). On the contrary, an increased number of macrophages was observed in cerulein-treated mice (Fig. 9). Furthermore, the number of macrophages were significantly increased by the G-CSF in cerulein-treated mice at week 7 (28.65±1.17 vs 19.55±1.23/HPF, p<0.001) and week 9 (18.1±1.67 vs 9.85±2.54, p = 0.003). These results suggested that the G-CSF stimulated macrophages might play a potential role in the regression of pancreatic fibrosis.

## Discussion

CP is often caused by chronic repetitive inflammation within pancreas, resulting in activation of the profibrotic cascades and pancreatic fibrosis. To date, the therapies are imperfect and many patients remain suffering from chronic pain, exocrine and endocrine insufficiency [1]. G-CSF is a hematopoietic mobilizer and has been applied to all types of leucopenia in the clinical setting. It has been shown that G-CSF can accelerate the regression of fibrosis in organs including heart, lung and liver [4]–[7]. However, whether it has the anti-fibrotic effects in pancreas is still unknown. In the present study, we demonstrated that G-CSF significantly reduced pancreatic fibrosis in a cerulein-induced CP mouse model.

In heart, Sato *et* *al.* demonstrated that G-CSF treatment after myocardial infarction reduces fibrosis in a swine ischemia/reperfusion model [6]. The pigs in the G-CSF group were injected with G-CSF subcutaneously (5.0 ug/kg/day) for 6 days after myocardial infarction and sacrificed after 4 weeks. The fibrotic area and collagen III mRNA expression of infarcted hearts was significantly reduced in the G-CSF group compared with control. In a rat isoproterenol-induced cardiac injury model, the administration of G-CSF (50 ug/kg/day) for 7 days significantly reduced the fibrotic area and cardiac hypertrophy [4]. In an aortic constriction cardiac injury mouse model, the G-CSF (300 ug/kg/day) was administrated subcutaneously in the first 4 days after a debanding procedure also reduced the cardiac fibrosis significantly [23]. Further analysis showed there were no direct effects of G-CSF on cardiac fibroblasts or enhancing the transdifferentiation of mobilized BM cells. However, the considerable infiltration of neutrophils and increased expression of IL-1β directly induced the expression of MMP-2 and MMP-9 in cardiac fibroblasts may be considered as the possible mechanism of fibrosis regression. In a bleomycin-induced lung injury mouse model, G-CSF was administered intraperitoneally at a continuous dose of 50 ug/kg for 5 consecutive days starting 1 day after bleomycin. It induced mobilization of BM cells to the lung, decreased collagen deposition and improved the survival [7]. In liver, Higashiyama *et* *al*. demonstrated large number of BM-derived cells migrated into fibrotic mice liver, some of which express MMP-13 and MMP-9 during the regression process. During the last 7 days before the final CCl_4_ injection, the administration of G-CSF at 100 µg/kg continuously for 7 days not only enhanced mobilization and homing of BM-derived cells, but also accelerated the regression of liver fibrosis [5]. In another radiation-induced liver fibrosis mouse model, the G-CSF (200 µg/kg/day) at day 7, 14, and 21 after liver-radiation injury also increased the migration of BM-derived cells and reduced the degree of liver fibrosis [24]. In our study, a cerulein-induced CP mouse model was used to test the anti-fibrotic effects of G-CSF in pancreas. The results were compatible with previous studies in other organs. The amount of collagen, fibrotic area and ColI were significantly decreased in G-CSF-treated mice sacrificed at week 9. Although the G-CSF did not change the number of myofibroblasts and BM-derived myofibroblasts, it significantly mobilized and enhanced BM cells migrating into fibrotic pancreas, suggesting the potential anti-fibrotic role of migrated BM cells.

Myofibroblasts are cells with pivotal roles in organ fibrosis. A growing body of evidence indicates that cells from BM can differentiate into myofibroblasts during inflammatory and fibrotic responses [8]. BM-derived myofibroblasts were also observed in experimental models of pancreatic fibrosis, accounting from 6.12% to 20.2% of α-SMA positive myofibroblasts [10], [11]. Furthermore, the BM-derived CD45+ColI+ fibrocytes were demonstrated in peripheral blood and fibrotic pancreatic tissues [13]. The roles of BM-derived myofibroblasts remain controversial. While some studies demonstrated the anti-fibrotic MMP-9 and MMP-13 were expressed in the BM-derived cells [5], [25], [26], others showed the expression of mRNA for procollagen α1 was observed in BM-derived cells indicating their fibrogenic potential [13], [27], [28]. In our study, the number of myofibroblasts and BM-derived myofibroblasts were not changed, excluding a functional activation of myofibroblasts and BM cells transdifferentiation caused by G-CSF.

The effects of G-CSF in the early stage of severe acute pancreatitis have been demonstrated in several animal models. The administration of 90 µg/kg G-CSF 1 hour before the induction of pancreatitis in rats increased the number of peritoneal-exudate neutrophils and circulating neutrophils without increasing the concentration of TNF-α, IL-6, and IL-1β [19]. In a cerulein-induced acute pancreatitis mouse model, the G-CSF (120 ug/kg) administration increased the number of neutrophils and improved expression levels of opsonin receptors on neutrophils [20]. These results suggested that G-CSF mobilized neutrophils during acute pancreatitis. Furthermore, the protective effects of G-CSF were also demonstrated on mice with severe acute L-arginine-induced pancreatitis [18]. In our study, the number of migrated BM cells was also increased in the fibrotic pancreas of G-CSF treated mice. Further analysis showed the migrated BM cells were mainly macrophages.

Macrophages have been considered to have both pro-fibrotic and anti-fibrotic effects depending on the stage of chronic injuries [29]. In liver, the depletion of macrophages during the active injury phase resulted in reduced scar and fewer fibroblasts. On the contrary, depletion of macrophages during the recovery phase led to a failure of matrix degradation. It has been shown that a population of BM cells predominates, which resemble classical macrophages but promote matrix degradation through secretion of collagenase and other MMPs [30]. In this study, we also demonstrated the increase of macrophages in the fibrotic pancreas of G-CSF-treated mice, suggesting that G-CSF may mobilize this specific population of macrophages and promote the regression of fibrosis.

One limitation of this study is that we performed whole BM transplantation but not a specific subtype of BM cells. The adult BM contains both haematopoietic and mesenchymal stem cells, it is unclear which type (or both) of BM cells stimulated by G-CSF contribute to the decrease of pancreatic fibrosis. Although the simultaneous regression of fibrosis and increased accumulation of macrophages occurred within the pancreas of G-CSF treated mice, the possible mechanisms responsible for this were not further investigated.

### Conclusion

To our knowledge, this study is the first to demonstrate that G-CSF can reduce the degree of fibrosis in a CP mouse model. The MMP-13 production from myofibroblasts was modestly increased by G-CSF *in* *vitro*. The number of myofibroblasts and BM-derived myofibroblasts were not changed by G-CSF. However the increased number of non-myofibroblastic BM-derived cells and macrophages was observed in the pancreata from CP mice treated with G-CSF, suggesting a potential anti-fibrotic role of G-CSF through the enhancement of these specific cells.

## Supporting Information

S1 Fig **The FACS analysis of circulating GFP(+) cells.** (A) The percentage of GFP(+) cells and GFP (−) cells of circulating leukocytes of each mouse in different groups. (B) The average percentage of circulating GFP(+) cells in each group.
(TIF)

S2 Fig **The IHC staining of CD3 of pancreas from cerulein-treated mice with or without G-CSF sacrificed at week 7 and week 9.** Spleen tissues were used as IHC positive control.
(TIF)

S3 Fig **The IHC staining of B220 of pancreas from cerulein-treated mice with or without G-CSF sacrificed at week 7 and week 9.** Spleen tissues were used as IHC positive control.
(TIF)

S1 Table **The number of mice in WT mice experimental groups.**
(DOC)

S2 Table **The number of mice in BM-transplanted mice experimental groups.**
(DOC)
