# Supplementary figures and images for: Granulocyte Colony-Stimulating Factor Reduces Fibrosis in a Mouse Model of Chronic Pancreatitis
Source: PLoS One. 2014 Dec 31;9(12):e116229. doi: 10.1371/journal.pone.0116229 (PMC4281240; doi:10.1371/journal.pone.0116229)

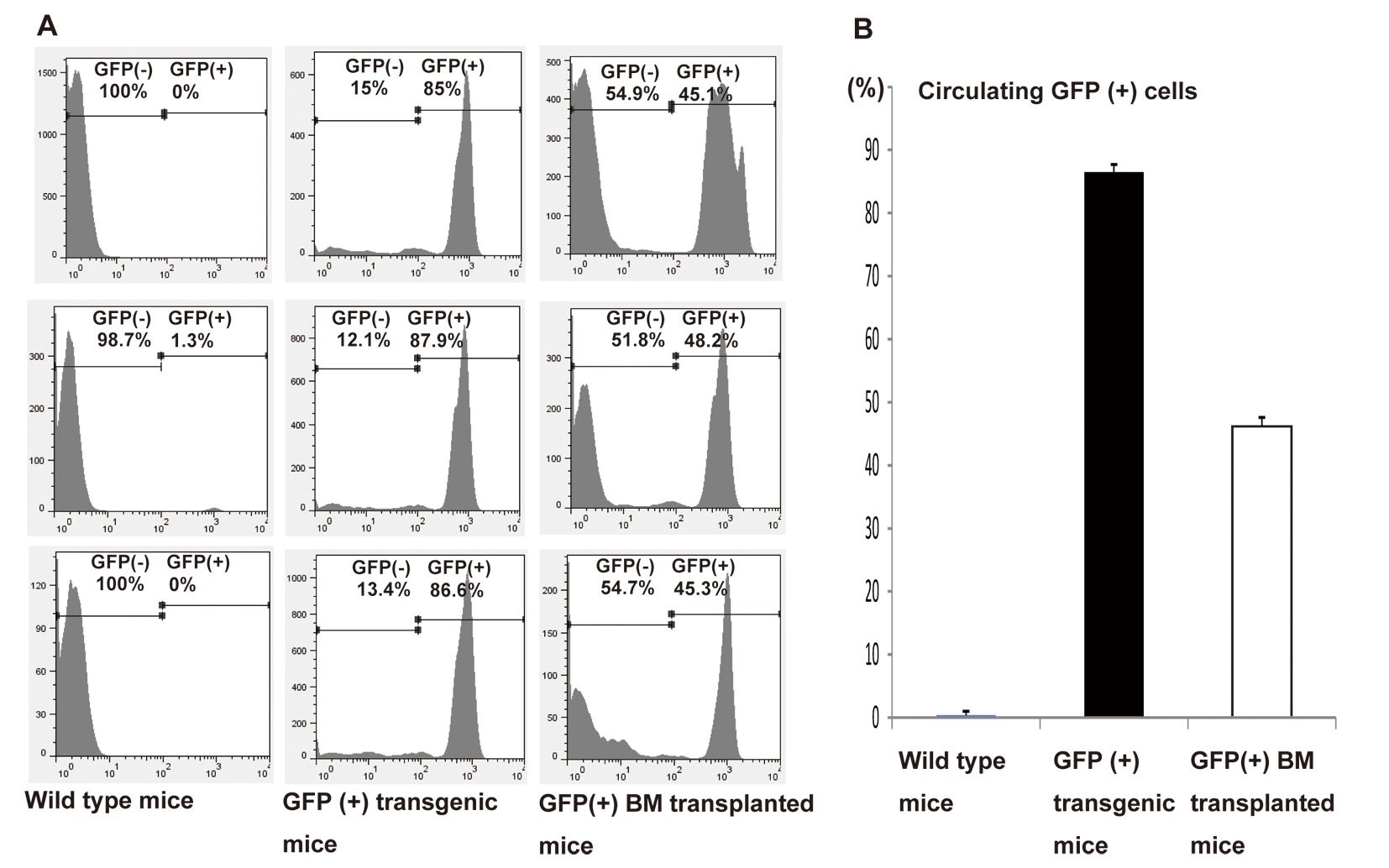

Supplement: S1 Fig — The FACS analysis of circulating GFP(+) cells. (A) The percentage of GFP(+) cells and GFP (−) cells of circulating leukocytes of each mouse in different groups. (B) The average percentage of circulating GFP(+) cells in each group. (TIF) [file pone.0116229.s001.tif]

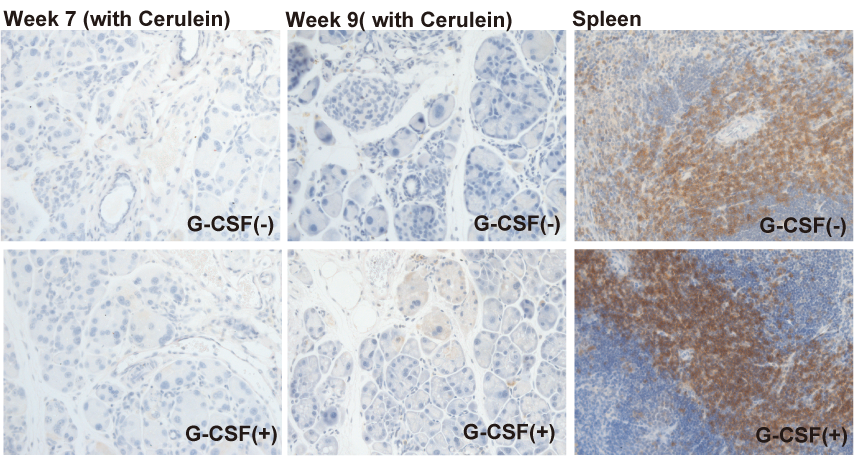

Supplement: S2 Fig — The IHC staining of CD3 of pancreas from cerulein-treated mice with or without G-CSF sacrificed at week 7 and week 9. Spleen tissues were used as IHC positive control. (TIF) [file pone.0116229.s002.tif]

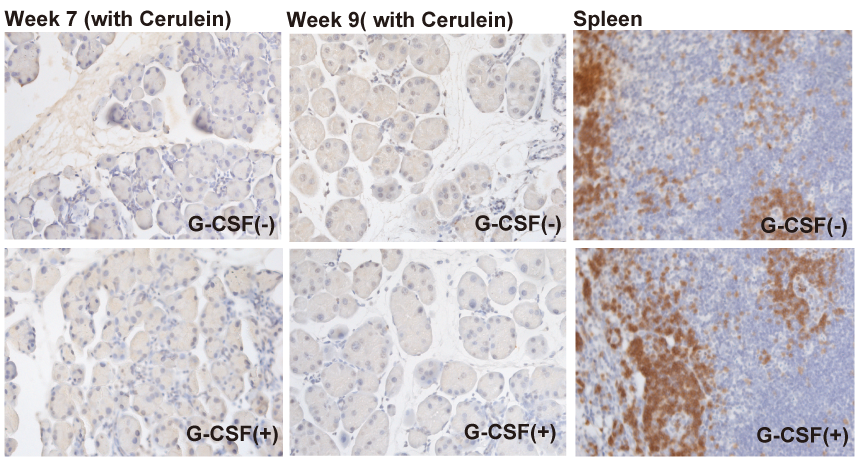

Supplement: S3 Fig — The IHC staining of B220 of pancreas from cerulein-treated mice with or without G-CSF sacrificed at week 7 and week 9. Spleen tissues were used as IHC positive control. (TIF) [file pone.0116229.s003.tif]
