# Supplementary material for: Granulocyte Colony-Stimulating Factor Reduces Fibrosis in a Mouse Model of Chronic Pancreatitis
Source: PLoS One. 2014 Dec 31;9(12):e116229. doi: 10.1371/journal.pone.0116229 (PMC4281240; doi:10.1371/journal.pone.0116229)
Supplement: S1 Table — The number of mice in WT mice experimental groups. (DOC) [file pone.0116229.s004.doc]

**Table S1**. The number of mice in WT mice experimental groups

| Mice group | W7 (N=18) | W9 (N=18) |
| --- | --- | --- |
| Control | N=3 | N=3 |
| Cerulein | N=6 | N=6 |
| G-CSF | N=3 | N=3 |
| Cerulein and G-CSF | N=6 | N=6 |
