# Supplementary material for: Granulocyte Colony-Stimulating Factor Reduces Fibrosis in a Mouse Model of Chronic Pancreatitis
Source: PLoS One. 2014 Dec 31;9(12):e116229. doi: 10.1371/journal.pone.0116229 (PMC4281240; doi:10.1371/journal.pone.0116229)
Supplement: S2 Table — The number of mice in BM-transplanted mice experimental groups. (DOC) [file pone.0116229.s005.doc]

**Table S2**. The number of mice in BM-transplanted mice experimental groups

| Mice group | W0 (N=3) | W7 (N=25) | W9 (N=25) |
| --- | --- | --- | --- |
| To assess re-established BM | N=3 |  |  |
| Control |  | N=3 | N=3 |
| Cerulein |  | N=9 | N=9 |
| G-CSF |  | N=4 | N=4 |
| Cerulein and G-CSF |  | N=9 | N=9 |
